# Should the current radiation protection paradigm and its recommendations be modified to make them more fit to protect the public in future nuclear emergencies?

**DOI:** 10.1093/rpd/ncae088

**Published:** 2024-11-14

**Authors:** James Mc Laughlin

**Affiliations:** School of Physics, University College Dublin, Belfield, Dublin 4, Ireland

## Abstract

The present radiation protection paradigm and its associated recommendations as developed by bodies such as the ICRP have performed very well over past decades both for those occupationally exposed to radiation and for the public in planned exposures. There is, however, growing evidence that the role played by this paradigm in the decision-making process to protect the public in nuclear emergencies in the past may have, unwittingly and unintentionally, caused more harm than good to some sections of the public. This seems to have been the case in the use of population evacuation as the principal protection response to the Chernobyl (1986) and Fukushima (2011) accidents. There is thus a need to develop improved guidelines or tools on how to apply radiation protection recommendations for the public compatible with the Principle of Justification in the event of any future major radiation emergencies. It can also be argued that the present radiation protection paradigm, with its emphasis primarily on the physical health detriments from radiation, should be more inclusive and needs to shift to a more holistic or total health approach than heretofore to include mental health effects associated with nuclear emergencies. For severe mental health effects, some of the consequences, such as suicide, can even be as or more severe than most physical detriments likely to be suffered by those affected.

## Introduction

Commencing with the accident in September 1957 at the Kyshtem/Mayak nuclear plant in the USSR and that at Windscale in England in October 1957, a number of other major accidents have since occurred at nuclear facilities throughout the world^**(**^^1^^,^  ^2^^)^. In terms of their reported impact on the lives of the general public, the most notable of these occurred at Chernobyl, USSR (1986) and at Fukushima, Japan (2011)^(3, 4)^.

Agencies concerned with radiation safety such as the IAEA classify incidents and accidents in nuclear power plants in terms of their significance or magnitude. Here, however, the term nuclear emergency is used in the generally understood sense of a sudden occurrence or accident at a nuclear facility with a potential to cause significant harm to both plant operatives and the general population living close to the affected plant. The protective actions that may be taken by the relevant authorities for nuclear emergencies are well described in the literature^(5)^. These can range from the issuing of thyroid blocking tablets to sheltering, to large-scale evacuation and to control of artificial radionuclide in foodstuffs. The action taken to destroy milk to protect children in particular from ingesting ^131^I used as a protective action by the UK authorities following the Windscale accident in 1957 is an early example of the latter^(2)^. Mass evacuations were the principal protective action used by the authorities to protect the public in the aftermath of the Chernobyl (1986) and Fukushima (2011) accidents^(3, 4)^. It has been both implicit and explicit that the use of any protective action should always be compatible with the Principle of Justification to ensure that the good achieved exceeds any harm than may consequently occur.

Any protective actions that can be used has associated advantages and disadvantages. In the case of ITB (Iodine Thyroid Blocking) to be effective, the blocking agent (usually Potassium Iodide tablets) ideally should be pre-distributed before the accident occurs and taken shortly before or after the accident. These requirements are difficult to comply with when faced with dealing with an emergency which, almost by definition, is usually unexpected and with rapidly changing characteristics sheltering in homes and other available buildings can be used as an effective short-term action in some situations. However, due to the inevitable difficulty of maintaining adequate supplies of uncontaminated food, water and medical supplies following a nuclear accident sheltering is not suitable as a long-term protective action. Restrictions on the use of food contaminated by artificial radionuclides were, for example, extensively used following the Chernobyl accident. These were applied not only locally but also in many countries far distant from the accident which were affected by the deposition of airborne contamination^(6)^. In keeping with the then international recommendations on dose limits, controls were strictly applied on the permitted levels of artificial radionuclides in meat (^137^Cs in particular) and other foodstuffs used for human consumption. In retrospect, this type of protective action aimed at averting small radiation doses had a negative effect on the socio-economic and psychosocial life of some food producing communities. An example of this occurred in the north of Sweden where the intervention level or limit for caesium in reindeer meat was set initially in May 1986 at 300 Bq kg^−1^. This low limit was raised to 1500 Bq kg^−1^ in June 1987, but the imposition of the initial limit had by then produced a major impact on the socio-economic and cultural life as well as on the mental health of the Sámi who are an indigenous community of traditional reindeer herders in the northern arctic regions of Scandinavia. The limits set in Norway, 600 Bq kg^−1^ (1986) and 6000 Bq kg^−1^ (1987), were not as strict as in Sweden and consequently had lessor impacts on the local Sámi communities there. The radiological health benefit of the controls set initially in Sweden is questionable, and it can be argued that they were not compatible with the Principle of Justification when the totality of their health impact is considered. Indeed, this was later partially acknowledged in 2002 when Lars-Erik Holm the then Director of SSI and ICRP Chairman (2005–09) made an unambiguous statement in the Swedish national press that ‘the 300-Bq kg^−1^ intervention level for reindeer meat was far too low’^(7)^.

## Dilemmas for the authorities

Nuclear accidents generally occur unexpectedly. Authorities are then faced with the urgent problem of making decisions to take protective actions in a rapidly changing situation. Notwithstanding the extensive scientifically based advice and recommendations from national emergency preparedness plans and international bodies such as the ICRP and the IAEA, the decisions of the relevant authorities, particularly in open democratic societies, can additionally be strongly influenced by political and socio-economic pressures. The political pressures on the authorities usually can be traced to public fear of the perceived risks to health from radiation exposure and to the activities and objectives of anti-nuclear protest groups. In addition to the rapidly changing conditions of an accident, accurate information on dose distributions due to contamination in the local environment may not be promptly available. Faced with such a complicated scenario, the decision-making authorities are faced with an unenviable task. The internationally formulated recommendations to carry out protective actions can become become somewhat academic in the rapidly changing conditions of an emergency. Post-accident analyses of the health impact of decisions taken to carryout large-scale evacuations of the public across all age groups following the Chernobyl and Fukushima accidents indicate that these actions caused more harm than good to some sections of the public^(8–13)^.

Detailed discussion of nuclear emergency evacuation and relocation plans are outside the scope and main focus of this paper. It is, however, useful to make some comments on the use of evacuation as a protective action as it presents the authorities with a number of dilemmas. While some form of evacuation as a protective action may be necessary and unavoidable in a rapidly changing emergency, it can also have negative impacts on the evacuees ranging from short-term direct health effects during the evacuation to long-term mental and physical health effects as well as on socio-economic life^(14)^. In spite of this some form of evacuation seems unavoidable. On the other hand, if potential emergency conditions develop within a nuclear power plant but are rapidly and successfully brought under full control with no releases to the environment, the authorities might decide that no mandatory evacuation of the public is considered necessary on radiological health grounds. Even in such a scenario, experience from the Fukushima accident strongly suggests that voluntary evacuations would occur with their concomitant impacts on mental health and on the socio-economic life of the region. Large-scale evacuations of the public took place following the accidents at Chernobyl (approx. 35 400 persons in the period 1986–92) and at Fukushima (approx. 118 000 persons in 2011). A number of major reports on these accidents state that the principal observed health effects in these populations were indirect nonphysical psychological/social effects arising from the processes of evacuation and relocation^**(**^^3^^,^  ^4^^,^  ^14^^)^. The impact on the mental health and socio-economic life of the evacuees has already been noted. As each disaster has its own unique set of characteristics, the response of the authorities to it will reflect these and also the polity and social mileu in which it occurs. Therefore, while there are valuable lessons to be learned from how the relevant authorities responded to the nuclear disasters at Chernobyl and Fukushima, caution should be exercised in expecting to find these lessons to be applicable in all national radiation preparedness strategies.

In the case of the Fukushima accident, comparative analyses of the prefecture morbidity and mortality statistical data bases for before and after the accident were carried out. On this basis, it has been estimated that the premature death rate or loss of life expectancy (LLE) in elderly evacuees was far in excess of either the short- or long-term estimated fatal cancer death rates in this cohort due to the direct effect of their exposure to radiation associated with the accident^(13, 15)^. Those most affected were the elderly with underlying medical conditions who were already resident in hospitals and care homes prior to the accident. Both their transportation and relocation in the evacuation process severely disrupted their essential medical and social care thereby contributing to their premature deaths. As an example, it has been estimated that the absolute risk of death caused by the evacuation of the elderly without their medical and other essential supports was ~60 deaths per thousand^(13)^. It has also been clearly shown that the magnitude of the LLE in the evacuated elderly was greatest for those evacuated rapidly or early in the emergency but declined for those evacuated in other later evacuation phases^(15)^. This finding highlights the need to develop specific evacuation plans for the vulnerable elderly to optimise or balance the trade-off between their evacuation and radiation risks. It should also be noted that numerous studies in recent years have found convincing evidence that various physical health effects such as diabetes and hyperlipidemia increased after the Fukushima disaster and are attributable to changes in lifestyle resulting from the disaster^(16)^. These studies also observed that indirect disaster-related deaths, diseases of the circulatory system and those of the respiratory system were also major causes of death^(17–20)^**.** The findings of these studies are further compelling evidence that limiting radiation protection of the public, following a significant nuclear powerplant accident, to considerations only of the carcinogenic aspects of radiation exposure can no longer be justified.

At present, accident preparedness strategies and protective actions taken by the relevant authorities to protect the public from the impact of nuclear accidents seem to be almost exclusively focussed on protecting against possible consequential direct physical health risks from ionising radiation such as fatal cancers. In keeping with this bodies such as the IAEA issue advice based on current ICRP recommendations on dose limits. Underpinning the ICRP radiation protection paradigm is its multidimensional concept of ‘Detriment’. The principal components of the detriment from radiation considered are ‘stochastic quantities such as the probability of attributable fatal cancer, the weighted probability of attributable non-fatal cancer, the weighted probability of severe heritable effects, and length of life lost if the harm occurs’^(21)^. The ICRP definition of detriment also uses the undefined term ‘total’ in relation to harm to health. This is in contrast to the clearer and more holistic approach of the WHO which states in its constitution that:

‘Health is a state of complete physical, mental and social well-being and not merely the absence of disease or infirmity’^(22)^.

Assessments of the total public health impact of the accidents at Chernobyl and Fukushima have shown that their impact on the psycho-social, mental health and economic life of the affected communities were quite severe^(3, 4)^. It is therefore appropriate that the radiation protection community should now debate if the present exclusive emphasis on the physical detriments from radiation should be broadened and modified to protect the public in nuclear emergencies also from negative impacts on their mental health and socio-economic well-being. It can be argued that dealing with these latter type of problems should be within the domain of radiation protection in particular when dealing with the impact of nuclear accidents on the public. To argue, otherwise is contrary to the ethics of radiation protection. Notwithstanding the complex changes in radiation protection recommendations that will be required if the current radiation protection paradigm were broadened, these should have little effect on most existing routine non-emergency radiation protection procedures. It could however be of considerable assistance to decision-makers in emergency situations to help them make decisions on protective actions more in keeping with the Principle of Justification than heretofore.

## Public fear of radiation and its influence in emergencies

In common with other actions taken by national authorities to protect the health and well-being of its citizens (e.g. controls on traffic, water and food quality, tobacco and alcohol use, etc.), controls on radiation exposure are usually a balance or trade-off between the benefit and possible harm of such controls as well as of their societal acceptability. For example, in spite of the unacceptable rate of road deaths in all countries, society reluctantly recognises that achieving a zero risk of injury or death due to road traffic cannot be achieved within the framework of present day socio-economic structures and transport technologies. In the case, however, of real or perceived risks from radiation, there seems to be an almost universal opinion in society that the authorities should have the unachievable objective of reducing the risks from accidental releases of radionuclides into the environment to zero.

Society in general does accept that the benefits of practical applications of radiation technologies in medicine and industry do involve some small risks and regulates them accordingly. It should, however, be noted that even in the case of beneficial medical applications, public fear of radiation can be present^(23–26)^. The origins of public fear of radiation is traceable back over a century ago to the injuries observed during the early use of X-rays and radioactive sources in medical diagnostics and radiotherapy. Indeed, the establishment of the IXRPC (International X-Ray and Radiation Protection Committee) in Stockholm in 1928, which can be viewed as the precursor of ICRP (established in 1950), was to deal with these health effects. Public fears of radiation increased considerably due to the atomic bombings of Hiroshima and Nagasaki in 1945 and the subsequent nuclear arms race by major powers. Even though a nuclear power plant designed to produce electricity cannot in any circumstance explode such as a nuclear bomb, some sections of the public perceive this to be possible. Fear or radiation is also amplified by the dominant role in radiation protection of the controversial linear no threshold dose-risk hypothesis, commonly called the LNT model. The LNT model can be traced back to the work in the 1920s of the geneticist Hermann Muller (Nobel Laureate Physiology/Medicine 1946). He observed heritable changes in the phenotype of fruit flies resulting from their irradiation by exceedingly high doses of X-rays which would be lethal to humans^(27)^. He interpreted these phenotype changes as due to changes in the genotype and he considered them as radiation-induced mutations. He also unequivocally stated, without supporting evidence, that the occurrence and frequency of these mutations was linearly related to the dose extrapolated ‘without a threshold down to zero dose’. In retrospect, this hypothesis was reasonable for high-dose exposures but highly speculative for low-dose exposures (≤100 mSv) where there was no experimental evidence then available. Equally importantly, there was no knowledge at that time of the various adaptive responses to radiation damage at low doses. Today there is, however, a large body of evidence available on adaptive responses at low doses which may protect irradiated organisms against cancer induction. Presently known adaptive responses range from apoptosis to immune system responses to the repair of DNA single and double strand breaks, etc. It should be noted that DNA alteration rates due to normal metabolic processes greatly exceed those arising from radiation damage at low doses.

How and why international radiation protection bodies such as the ICRP and IAEA adopted the LNT model which underpins their recommendations is a complex story involving both scientific and geo-political considerations as well as regulatory convenience and is outside the scope of this short paper. The use by the ICRP of the contentious LNT model as a conservative protective measure in combination with the Precautionary Principle may contribute to a tendency to overregulate at low doses thereby, however unwittingly and unintentionally, amplifying the public perception of risks at low doses. Public response to the recent discharges into the Pacific Ocean of ALPS (Advanced Treatment Processing System) treated water containing very low concentrations of tritium from storage tanks at the Fukushima Daiichi Nuclear Power Plant is a good example of this. These discharges commenced in August 2023 and it is reported that the tritium content of the treated water at the point of discharge is nominally about 1500 Bq l^−1^. This is 1/40 of the Japanese government safety standard of 60,000 Bq l^−1^ and about 1/7 of the WHO guideline of 10,000 Bq l^−1^ for drinking water quality^(28)^. However, this setting of the tritium concentration of the discharged water well below the existing standards may actually help to increase public anxiety as it may give rise to an incorrect public perception that the existing official standards were originally set too high for safety. If this is found to be the case, it could be argued that a strategy to discharge water at a tritium concentration well below accepted safety standards may be an example of the law of unintended consequences in operation. Expert scientific opinion both in Japan and internationally is that the radiological impact to humans and the environment due to the these discharges is miniscule^**(**^^29^^,^  ^30^^)^. Due to the large amount (~1.3 million tons) of treated water to be discharged, setting the level at 1500 Bq l^−1^ will prolong the discharge operation for up to 30 years. Apart from radiological health considerations, local coastal communities are concerned that a 30 year discharge plan time frame will severely handicap local industries and impede post disaster socio-economic recovery^(31)^**.** In addition to some sections of the public both in Japan and in neighbouring countries, there has been considerable reluctance to consume sea food harvested from Japanese waters. Sea food is a major component of the Japanese diet and its dietary health benefits are substantial and well recognised. Prolonged significant reduction in sea food consumption could result in dietary changes to less healthy foods with associated health risks far in excess of the negligible and unverifiable radiological risks that might result from consuming seafood containing trace amounts of artificial radionuclides from the Fukushima accident. This indicates that science-based official public radiation health communication programs need to be considerably improved to counteract the negative effects on public perceptions caused by the misinformation on radiation matters widely available to the public.

In the present context of nuclear emergencies, fear of radiation may have been a significant factor in influencing protective action decisions by the relevant authorities in the immediate aftermath of emergencies in recent decades. If this opinion is even partially correct, then the global radiation protection community has been remiss and ineffective in its ethical task of informing the general public of the scientifically known facts of the health effects of radiation exposure in both high and low dose ranges. As already noted, the resulting information vacuum has often been filled with misinformation from non-expert sources and also even intentional disinformation from dubious sources. It is generally accepted that improved and effective communication between the public and experts on radiation matters is needed to counteract this. To date, the traditional model of radiation risk communication has been largely a unidirectional information flow from the experts to the public. This should shift more to a citizen-centred or dialogue-based approach which would benefit not only the public but also the experts and the authorities in emergency preparedness planning^(32, 33)^. Fortunately significant nuclear power plant emergencies are rare events. This should afford the authorities the opportunity during normal non-disaster periods to include or improve upon basic scientific knowledge on radiation and its health effects in national school curriculum topics on environmental and health studies. Apart from the general educational advantages of this, having a more objectively informed population on radiation matters would be of assistance to national emergency preparedness planning**.**

## Conclusion

Protecting the general population in the event of an emergency at a nuclear power plant presents the authorities with many dilemmas. Decisions to take appropriate protective actions in emergencies must be made quickly in rapidly changing and unpredictable conditions with partial or otherwise incomplete information available on the levels of contamination and associated doses. In addition to such a situation, the fear in the population living close to the accident can place intolerable pressure on the authorities to take effective protection action quickly. For the public evacuation, either voluntary or mandatory, can be perceived as desirable. Analyses, however, of the health effects, both physical and mental, consequent to large-scale evacuations of the general population by the relevant authorities following the accidents at nuclear power plants in Chernobyl and Fukushima show that evacuation is not always compatible with the Principle of Justification. In particular in the case of the Fukushima accident, the excess premature deaths and LLE of the cohort of evacuated elderly citizens, due mainly to the disruption of their medical support requirements, were far in excess of any expected early or late deaths due to the direct effects radiation exposure. This was clearly not in keeping with the principle of justification at least for that cohort. In addition to physical health effects, a significant public health impact of these two accidents was in the area of mental health, which the present international recommendations do not adequately address. Therefore, as an essential component of future emergency preparedness, there is an urgent need for the international radiation protection community to develop tools and strategies to protect the public in future radiological emergencies not only from the direct physical health effects of radiation exposure but also from psycho-social and socio-economic effects of such emergencies.

In keeping with the ongoing process by the ICRP to review and revise its system of radiological protection to keep its recommendations fit for purpose, there is an obvious need to discuss and debate the above issues in the radiation protection community. At the time of writing, this is of particular urgency as there is already clear evidence of an increased risk of radiation emergencies happening at nuclear power plants in the Ukraine. This possibility is mainly due to the total loss of off-site electricity supplies to these power plants that has occurred on a number of occasions arising from military actions since March 2022. Accordingly, the IAEA has issued a number of regular warnings on the vulnerability of the safe operation of these plants in these circumstances^(34)^.
